# Epidemiological profile of COVID-19 in patients with prostate cancer undergoing androgen deprivation therapy at a Brazilian Cancer Center

**DOI:** 10.31744/einstein_journal/2023AO0273

**Published:** 2023-10-10

**Authors:** Isabela Granato Travalini, Lucas Bonachi Vergamini, Ivan Leonardo Avelino Franca e Silva, Pedro Caruso, Fernanda Monteiro Orellana, Maria Paula Curado, Stênio de Cássio Zequi

**Affiliations:** 1 Universidade Nove de Julho São Paulo SP Brazil Universidade Nove de Julho , São Paulo , SP , Brazil .; 2 A.C.Camargo Cancer Center São Paulo SP Brazil A.C.Camargo Cancer Center , São Paulo , SP , Brazil .; 3 Universidade Federal de São Paulo São Paulo SP Brazil Universidade Federal de São Paulo , São Paulo , SP , Brazil .

**Keywords:** COVID-19, SARS-CoV-2, Coronavirus infections, Prostatic neoplasms, Androgens, Antineoplastic agents, hormonal

## Abstract

**Objective:**

To describe the epidemiological aspects of COVID-19 in patients with prostate cancer who received androgen deprivation therapy and those who did not.

**Methods:**

We retrospectively analyzed the medical records of patients with prostate cancer undergoing androgen deprivation therapy and those who did not undergo androgen deprivation therapy. These patients were treated at the *A.C.Camargo Cancer Center* between March 2020 and March 2021.

**Results:**

Of the 78 patients with prostate cancer and positive RT-PCR test results, 50% were undergoing androgen deprivation therapy, and 49% were experiencing a non-metastatic biochemical relapse. Of these, 80.6% were symptomatic on the day of examination compared to 97.2% in the Control Group. A total of 82.1% of the patients receiving androgen deprivation therapy required hospitalization, with 30.8% admitted to the intensive care unit compared to 21.6% in the Control Group. There was no statistically significant difference in the use of a high-flow oxygen cannula, the need for orotracheal intubation and mechanical ventilation, the need for dialysis, multiple organ failure, or death. A significant difference was found between the groups in terms of the average length of stay in the intensive care unit.

**Conclusion:**

Androgen deprivation therapy was not associated with protective factors or potential treatments in patients with prostate cancer and COVID-19. Although the number of patients analyzed was limited, and there may have been a selection bias, this is a unique study that cannot be expanded or replicated in similar (unvaccinated) populations.

## In Brief

Older individuals with cancer constitute a high-risk group for COVID-19. Entry of the virus into cells occurs through the binding of the S protein with angiotensin-converting enzyme 2, which is mediated by the *TMPRSS2* gene and regulated by androgen receptors. Androgen deprivation therapy in patients with prostate cancer inhibits *AR-TMPRSS2* interactions, which in turn inhibits the aggressiveness of the infection.

## Highlights

We were unable to prove an association between the use of androgen deprivation therapy and a reduction in factors associated with worse clinical outcomes.

Most of the data presented show a tendency to favor the outcomes of patients who do not undergo androgen deprivation therapy, which can be explained by the fact that, in general, their clinical conditions are better and their performance status scores are lower than those of patients who undergo androgen deprivation therapy.

Abstract presented to the oncology department of *A.C.Camargo Cancer Center* as a conclusion of the Scientific Initiation.

## Visual Abstract

**Figure:**
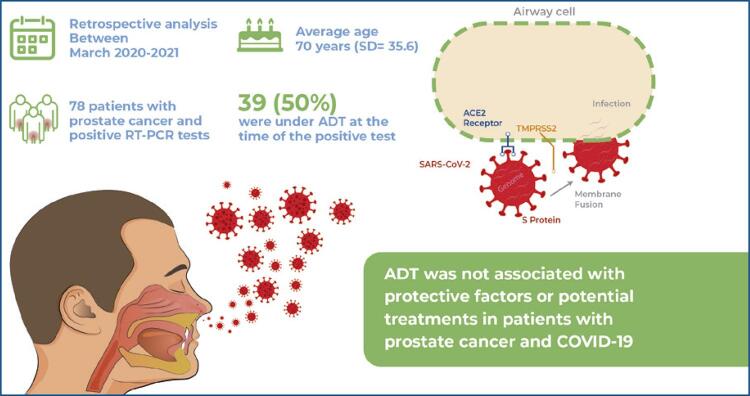

## INTRODUCTION

Several cases of human infections related to severe acute respiratory syndrome have been reported in Wuhan City, Hubei Province, China, between late 2019 and early 2020. ^( 1 )^ The disease, coronavirus disease 2019 (COVID-19), was quickly classified as a pandemic, and the pathogen was identified as a novel coronavirus, severe acute respiratory syndrome coronavirus 2 (SARS CoV-2), which is closely related to severe acute respiratory syndrome coronavirus (SARS-CoV) and Middle East respiratory syndrome coronavirus (MERS-CoV). ^( 2 )^

Although any age group is susceptible to contracting the disease, the first epidemiological survey identified the following risk factors for developing more severe forms: ^( 3 )^ advanced age, male sex, smoking, and comorbidities (such as obesity, diabetes, hypertension, and respiratory diseases) and ^( 4 )^ a positive history of malignant neoplasm. ^( 5 )^

The expression of the *TMPRSS2* gene has been associated with cases of prostate cancer (PC) and COVID-19. This enzyme belongs to the family of transmembrane serine protease type II (TMPRSS), is involved in several physiological and pathological processes in the body, and is considered a critical factor for the viral infection of cells. ^( 6 )^ Recent studies have reported that the entry of SARS-CoV-2 into the cell is mediated by angiotensin-converting enzyme-2 (ACE2), followed by the cleavage of peptide bonds of the S protein by the action of TMPRSS2, allowing the fusion of viral particles and cell membranes. ^( 4 , 6 )^

In cases of PC (localized or metastatic), TMPRSS2 is highly expressed, and its transcription is regulated by androgen receptors (AR) in both prostate and lung tissues. ^( 6 )^ The androgen-dependent regulation of TMPRSS2 expression in the lungs may explain the increased susceptibility of men to severe SARS-CoV-2 infections compared to women. ^( 4 )^

Androgen deprivation therapy (ADT), performed by castration (chemical or surgical) or by the administration of anti-androgen medications, is the mainstay of the treatment of advanced PC since the suppression of testosterone or blocking its connection to the AR (in the case of anti-androgens) results in fewer activated ARs and, consequently, a reduction in the transcription of the TMPRSS2 gene. ^( 4 )^

Montopoli et al. ^( 4 )^ showed that patients with PC who underwent ADT had a significantly lower risk of infections and diseases by SARS-CoV-2. Therefore, we evaluated whether patients undergoing ADT for the treatment of PC had more favorable outcomes than patients with PC who did not undergo ADT when they developed SARS-CoV-2 infection.

## OBJECTIVE

To investigate the pathogenicity and compare the complication rates of COVID-19 in patients with prostate cancer undergoing androgen deprivation therapy with those in patients with prostate cancer not undergoing this treatment.

## METHODS

We retrospectively analyzed the medical records of patients with a diagnosis of PC and SARS-CoV-2 infection, confirmed by positive reverse transcriptase polymerase chain reaction (RT-PCR) testing, who were hospitalized between March 2020 and March 2021 for COVID-19. Patients were included after signing an informed consent form. This study was approved by the Research Ethics Committee of *A.C.Camargo Cancer Center* (ACCCC) (CAAE: 42456820.0.0000.5432; #3037/21).

The inclusion criteria were patients over 18 years of age being treated at the ACCCC with a positive RT-PCR test for COVID-19 between March 1, 2020, and March 1, 2021. The exclusion criteria were as follows: suspected cases admitted without a confirmatory test for COVID-19 infection and patients who could not be followed up.

To identify cases with a positive RT-PCR test for COVID-19, we used the databases of the Hospital Infection Control Service and the COVID-19 Crisis Committee. Data from medical records under the International Classification of Diseases (ICD) “C-61” were analyzed to identify patients with PC.

The patients included in the study were divided into two groups based on the type of hormone therapy used: patients undergoing ADT (Group I) and those not undergoing ADT (Group II). These groups were compared in terms of clinical outcome and evolution, considering the following: undergoing ADT on the day of the COVID-19 test; need for hospitalization; length of stay (days) in hospital; need for intensive care unit (ICU); length of ICU stay (days); need for the use of high-flow oxygen cannula, orotracheal intubation (OTI), and mechanical ventilation (MV); need for dialysis; multiple organ failure; and death.

Statistical evaluation of the strength of the association between COVID-19 and the different groups was performed by calculating the odds ratio (OR). The χ ^2^ test was used to compare the frequencies. Statistical significance was set at p<0.005.

## RESULTS

**Figure 1:**
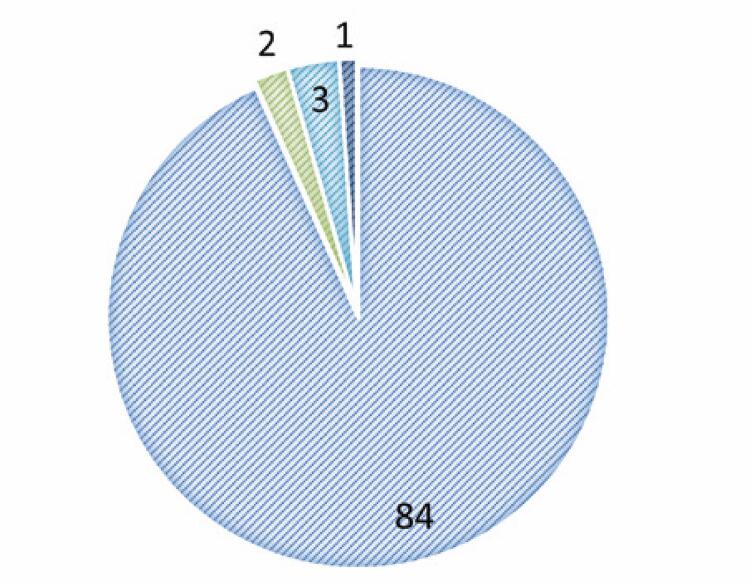
Total patients analyzed

**Figure 2:**
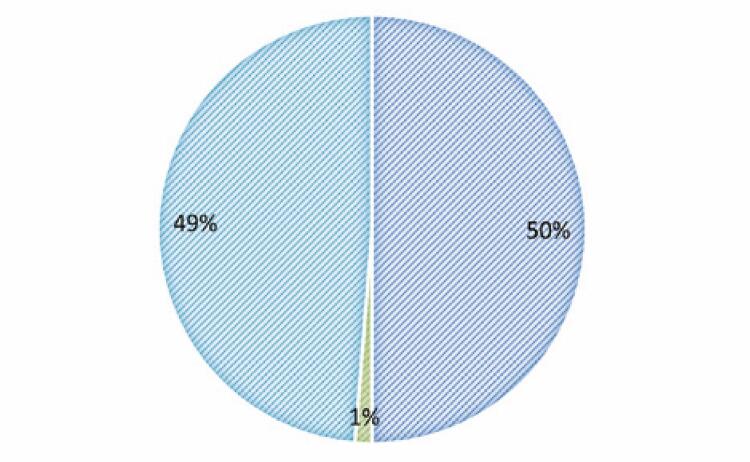
Comparative analysis of patients under androgen deprivation therapy

We identified 84 PC patients with positive COVID-19 RT-PCR results. Of these patients, two were excluded from the study because they had prostate sarcoma and four because they did not sign the informed consent form. The remaining 78 patients ( Figure 1 ) were included in the analysis, and their demographic characteristics are shown in table 1 . Of the 78 patients, 50% underwent ADT, and 49% did not under hormone therapy. The data of one patient could not be determined owing to inconsistencies ( Figure 2 ). The average patient age was 70 years (SD= 35.6), with an initial median PSA of 8.74 (SD= 72.9).

**Table 1:** Demographic data of the population studied, duration of hormone therapy and length of stay in hospital

Variables	Median	Standard deviation
Age (years)	70	35.06
BMI (Body mass index)	27.51	4.76
Initial PSA	8.74	72.92
ADT time (months)	24	36.74
Hospitalization time (days)		
Total	9	9.29
ICU	4	10.44

ADT: androgen deprivation therapy; ICU: intensive care unit; BMI: body mass index; PSA: prostate specific antigen.

Based on the biopsy findings ( Table 2 ), 10% of the patients had prostate neoplasms graded by the International Society for Uropathology (ISUP) as follows: ISUP 1, 7%; ISUP 2 or 3, 42.8%; and ISUP 4 or 5, 47.2%. Regarding disease status, only 2.6% were under active surveillance, 32.9% were cured after primary treatment, 36.9% had a non-metastatic biochemical relapse, 15.8% were metastatic after primary treatment, 11.8% were already metastatic at diagnosis, and 3% were castration-resistant ( Table 3 ).

**Table 2:** Study population variables, including the type of androgen deprivation therapy used and ISUP by prostate biopsy

Variables	n (%)
Biopsy ISUP	
1	7 (10)
2-3	30 (42.8)
4-5	33 (47.2)
Type of ADT	
First line	26 (33.8)
Second line	10 (13)
Adjuvant	2 (2.6)
Did not use	38 (50.6)

ISUP: International Society of Urological Pathology; ADT: androgen deprivation therapy.

**Table 3:** Disease status in the population studied

Disease status	n (%)
Active surveillance	2 (2.6)
Cured after primary treatment	25 (32.9)
Non-metastatic biochemical relapse	28 (36.9)
Hormone-sensitive	24 (31.6)
Castration-resistant	4 (5.3)
Metastatic post-treatment	12 (15.8)
Hormone-sensitive	6 (7.9)
Castration-resistant	6 (7.9)
Metastatic “again”	9 (11.8)
Hormone-sensitive	6 (7.9)
Castration-resistant	3 (3.9)

The vast majority of patients undergoing ADT were receiving drug regimen, and only one patient in the group underwent bilateral orchiectomy. In this group, 66.6% were on first-line hormone therapy and 25.6% were on second-line therapy.

As shown in table 4 , 80.6% of the patients in group I (under ADT) were symptomatic of COVID-19 on the day of the examination compared to 97.2% in the Control Group (p=0.055). Of the patients undergoing ADT, 82.1% required hospitalization owing to viral infection compared to 62.2% in the Control Group. However, this finding was not statistically significant (p=0.093). Of the patients who underwent ADT, 30.8% were admitted to the ICU compared with 21.6% in the Control Group (p=0.052).

**Table 4:** Variables comparing the exposure and Control Groups

	Group receiving ADT %	Control Group %	p value
Symptoms on examination day	80.6	97.2	0.055
Hospital admission	82.1	62.2	0.093
ICU admission	30.8	21.6	0.052
High-flow oxygen cannula	68.4	44.7	0.064
Orotracheal intubation and mechanical ventilation	8.3	18.4	0.310
Multiple organ failure	25	7.9	0.093
Death	30	10.5	0.056

ADT: androgen deprivation therapy; ICU: intensive care unit.

Regarding other findings during hospitalization, there were no statistically significant differences between the two groups in the following: the use of high-flow oxygen cannula (68.4% ADT Group *versus* 44.7% Control Group; p=0.064), need for orotracheal intubation and mechanical ventilation (8.3% ADT Group *versus* 18.4% Control Group; p=0.310), need for dialysis (only one patient in the Control Group required it) multiple organ failure (25% ADT Group *versus* 7.9% Control Group; p=0.093) and death (30% ADT Group *versus* 10.5% Control Group; p *=* 0.056).

Regarding the total length of stay, there was no association between the use of ADT and an increase in the length of hospital stay. The average length of stay for patients receiving ADT was 10 days (SD= 7.5), while for the Control Group, it was 8 days (SD= 11.2). However, a difference was found between the groups in the median length of time in the ICU, rejecting the null hypothesis when we compared ADT for 2.5 days (SD= 3.2) to the Control Group 26 (SD= 10.1).

## DISCUSSION

Since the World Health Organization (WHO) declared a state of Public Health Emergency of International Concern (PHEIC) and characterized the disease caused by the new coronavirus as a pandemic, ^( 1 )^ an explosion of ideas and hypotheses began to emerge aimed at treating or preventing the disease aggravations. ^( 7 )^

One of the main forms of treatment for patients with more advanced stages of PC is ADT through castration (chemical or surgical) alone or in combination with the administration of anti-androgens. ^( 4 )^ This type of treatment suppresses the levels of TMPRSS2 and the production of testosterone in the body to block the binding of androgens to their receptors. ^( 4 , 6 )^

TMPRSS2 is considered a critical factor in the entry and hosting of the virus that causes SARS-CoV-2 in human cells. ^( 6 )^ The *TMPRSS2* gene allows the fusion of viral particles and cell membranes by cleaving the peptide bonds between the S protein and ACE2. ^( 4 , 7 )^

Studies carried out during this period suggested that ADT performed on men with PC served as a protective or therapeutic factor, as they had a decrease in viral transmission, lower risk of infection, and lower mortality rates caused by COVID-19. ^( 8 )^ This effect was attributed to a decrease in *TMPRSS2* transcription and testosterone levels. ^( 7 )^ To test this hypothesis, several studies have evaluated the outcomes of COVID-19 infection in patients with PC who were undergoing ADT compared to those who were not undergoing this type of treatment. ^( 7 - 9 )^

Although we expected that the use of ADT could reduce infection, hospitalization, ICU admission, and mortality risk in patients with a positive history of COVID-19 infection, ^( 7 , 9 )^ such a relationship could not be established. ^( 7 - 9 )^ One possible explanation is that patients with PC who receive this type of treatment usually suffer from more comorbidities, advanced disease, and a higher risk of overall mortality. ^( 7 )^

The authors of an Italian study ^( 4 )^ concluded that the rates of COVID-19 infection were five times lower among those who underwent ADT than among those who did not undergo this type of treatment. ^( 4 )^ However, the study was performed at a population level with a high rate of COVID-19 cases. When examining a cohort of men with PC undergoing ADT, it was observed that they were less likely to report COVID-19 compared to those not undergoing ADT (4/5273 cases *versus* 114/37 161, OR *=* 4.05, 95% confidence interval (95%CI) 1.55-10.59, p *=* 0.00043). ^( 8 , 9 )^ Furthermore, tests for COVID-19 may have been less available at the beginning of the pandemic in Italy compared to the period when the study was conducted at our institution. The idea that ADT serves as a form of treatment or prevention for COVID-19 infection in people with PC, proposed by Montopoli et al. ^( 4 )^ seems unlikely, as there are not enough data to prove the theory. ^( 8 , 9 )^

Currently, the dissemination of vaccines and the development of new drugs have contributed to a significant reduction in complications and mortality rates among hospitalized patients. ^( 10 )^ As a result, there is less incentive for the use of ADT, as it is a high-cost drug that is associated with several side effects (metabolic syndrome, abdominal obesity, osteopenia and sexual and psychological dysfunctions), and its use outside the context of prostate cancer is not justified. ^( 8 , 9 )^

Our study was unable to prove an association between the use of ADT and a reduction in the factors associated with worse clinical outcomes or a reduction in the average length of hospital or ICU stay. Most of the data presented show a tendency to favor the outcomes of patients who do not undergo ADT. This can be attributed to the fact that, in general, their clinical conditions are better, and their performance status scores are lower than those of patients who undergo ADT. However, this study did not consider these variables.

As can be observed, the limited number of cases may have hindered the detection of statistically significant differences regarding patients undergoing ADT apparently having a longer hospital stay, increased use of a high-flow cannula, and more multiple organ failure. These patients probably had more comorbidities, a poorer prognosis, and were candidates for palliative and comfort therapies, rather than invasive measures such as OTI, which justifies the lower rate of OTI and invasive procedures in these patients.

This study has some limitations that are intrinsic to its retrospective nature. Additionally, data on comorbidities, such as performance status scores, hypertension, diabetes, and asthma, were not collected. Thus, the small sample size negatively affected the statistical analysis. Nevertheless, this is a representative sample of daily practice in national oncology centers.

## CONCLUSION

This study was unable to establish whether androgen deprivation therapy served as a protective factor or a potential treatment for patients with prostate cancer infected with the new coronavirus. Despite the limited number of patients analyzed and the possibility of selection bias, this is a unique study that cannot be expanded or replicated in similar (unvaccinated) populations.
